# Complete Genome Sequence of Pseudomonas Phage Zikora

**DOI:** 10.1128/MRA.00489-21

**Published:** 2021-07-29

**Authors:** Grace Chiemeka Ezemokwe, Francis Maji Agwom, Ijeoma Okoliegbe, Francis Obiora Okonkwo, Anayochukwu Chibuike Ngene, Nanko Gimba, Oluwatoyin Ruth Morenikeji, Abiodun Egwuenu, Chinelo Henrietta Okonkwo, John Chinyere Aguiyi, Nnaemeka Emmanuel Nnadi, Grégory Resch

**Affiliations:** a African Centre of Excellence in Phytomedicine Research and Development, University of Jos, Jos, Plateau State, Nigeria; b Department of Pharmaceutical and Medicinal Chemistry, Faculty of Pharmaceutical Sciences, University of Jos, Jos, Plateau State, Nigeria; c Institute of Dentistry, University of Aberdeen, Aberdeen, United Kingdom; d Department of Biochemistry, Faculty of Natural and Applied Sciences, Plateau State University, Bokkos, Plateau State, Nigeria; e Department of Microbiology, College of Natural Sciences, Michael Okpara University of Agriculture, Umudike, Abia State, Nigeria; f Antimicrobial Resistance Programme, Nigeria Centre for Disease Control, Abuja, Nigeria; g Department of Pharmacology and Toxicology, University of Nigeria, Nsukka, Enugu State, Nigeria; h Department of Microbiology, Faculty of Natural and Applied Sciences, Plateau State University, Bokkos, Plateau State, Nigeria; i Centre for Research and Innovation in Clinical Pharmaceutical Sciences, Lausanne University Hospital, Lausanne, Switzerland; DOE Joint Genome Institute

## Abstract

Pseudomonas aeruginosa is a major pathogen in humans and other animals, frequently harboring mechanisms of resistance to commonly used antimicrobials. Here, we describe the isolation of Pseudomonas bacteriophage Zikora. The full 65,837-bp genome was annotated and demonstrates similarity to *Pbunavirus* phages, making Zikora a new member of this genus of the *Myoviridae* family.

## ANNOUNCEMENT

The emergence of multidrug-resistant (MDR) Pseudomonas aeruginosa infections is becoming a global concern and has not spared Nigeria (1). P. aeruginosa is a Gram-negative opportunistic organism found in soils and aquatic environments (2). It has the capacity to cause a wide array of life-threatening acute and chronic infections, particularly in patients with compromised immune defenses (3).

**FIG 1:**
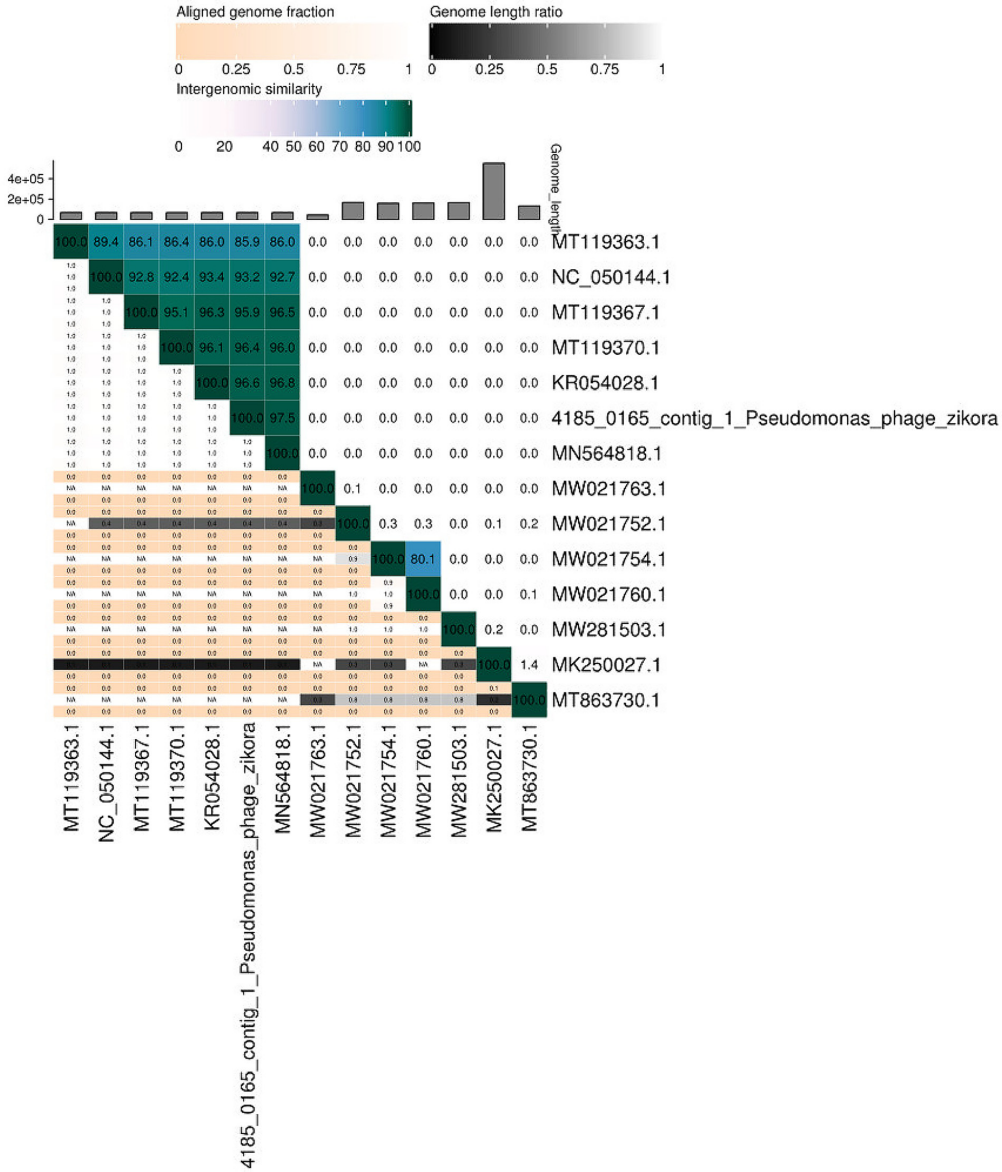
Classification of Pseudomonas phage Zikora by genome comparison using VIRIDIC (http://rhea.icbm.uni-oldenburg.de/VIRIDIC).

Zikora is a temperate bacteriophage that was isolated from sewage water from a hospital environment in Jos, Plateau State, Nigeria (latitude, 9°55′42.56″N; longitude, 8°53′31.63″E), in May 2020. It has been propagated using P. aeruginosa strain ACE015 as a host and the classic double-layer agar (DLA) method, as described previously (4). Genomic DNA was extracted from single large clear plaques and purified using the modified Promega Wizard DNA cleanup system shotgun library preparation protocol (5), prepared at Eurofins Genomics (Konstanz, Germany) as Illumina libraries using a self-established and validated protocol based on the NEBNext Ultra II DNA library preparation kit for Illumina, and sequenced at Eurofins Genomics on an Illumina NovaSeq 6000 system with an S2 flow cell. The Illumina Consensus Assessment of Sequence and Variation (CASAVA) software was used to perform a quality analysis of the 5,176,773 paired-end 150-bp reads obtained, and no additional adapter trimming was performed. Reads were assembled into a single contig with 18,274× average coverage using PATRIC (https://www.patricbrc.org) and Unicycler (6). Automated genome annotation was performed with Prokka v1.12 (7). ARAGORN (8) and tRNAscan-SE v2.0 (9) were used to search for tRNAs, and the life cycle of Zikora was determined using PHACTS (10). Phage termini and the packaging mechanism were determined using PhageTerm (11). A Web-based megaBLAST search was performed on the assembled contig using default settings to identify the closest related phages (https://blast.ncbi.nlm.nih.gov/Blast.cgi). VIRIDIC (12) was used to determine the species boundaries of Zikora (Fig. 1).

The complete genome of Zikora was obtained as a single contig of 65,837 bp with a G+C content of 54.88%. A total of 92 open reading frames (ORFs) and no tRNAs were predicted. At the nucleotide level, the closest neighbors of Zikora were found to be the pbunavirus Pseudomonas phage DRL-P1 (GenBank accession number MN564818) (97.77% identity with 100% coverage), phage DL52 (GenBank accession number KR054028) (97.38% identity with 100% coverage), and phage misfit (GenBank accession number MT119367) (95.63% identity with 100% coverage). Regarding its close identity with other pbunaviruses, i.e., a genus of the *Myoviridae* family of phages, Zikora was classified as a *Myoviridae* member by VIRIDIC. PhageTerm determined Zikora to package DNA by the classic headful mechanism (13). Zikora is a new temperate phage of interest since it extends the *Myoviridae* family, *Pbunavirus* genus, for which only 27 members are currently listed by the International Committee on Taxonomy of Viruses (ICTV) (https://talk.ictvonline.org).

### Data availability.

The genome sequence and associated data for phage Zikora were deposited under GenBank accession number MW557846, BioProject number PRJNA693824, BioSample number SAMN17478038, and SRA number SRX11023225.

## References

[1] Olowo-Okere A, Ibrahim YKE, Nabti LZ, Olayinka BO. 2020. High prevalence of multidrug-resistant Gram-negative bacterial infections in northwest Nigeria. Germs 10:310–321. doi:10.18683/germs.2020.1223. 33489946 PMC7811856

[2] Gellatly SL, Hancock RE. 2013. *Pseudomonas aeruginosa*: new insights into pathogenesis and host defenses. Pathog Dis 67:159–173. doi:10.1111/2049-632X.12033. 23620179

[3] Moradali MF, Ghods S, Rehm BH. 2017. *Pseudomonas aeruginosa* lifestyle: a paradigm for adaptation, survival, and persistence. Front Cell Infect Microbiol 7:39. doi:10.3389/fcimb.2017.00039. 28261568 PMC5310132

[4] de Melo ACC, da Mata Gomes A, Melo FL, Ardisson-Araújo DMP, de Vargas APC, Ely VL, Kitajima EW, Ribeiro BM, Wolff JLC. 2019. Characterization of a bacteriophage with broad host range against strains of *Pseudomonas aeruginosa* isolated from domestic animals. BMC Microbiol 19:134. doi:10.1186/s12866-019-1481-z. 31208333 PMC6580649

[5] Summer EJ. 2009. Preparation of a phage DNA fragment library for whole genome shotgun sequencing. Methods Mol Biol 502:27–46. doi:10.1007/978-1-60327-565-1_4. 19082550

[6] Wick RR, Judd LM, Gorrie CL, Holt KE. 2017. Unicycler: resolving bacterial genome assemblies from short and long sequencing reads. PLoS Comput Biol 13:e1005595. doi:10.1371/journal.pcbi.1005595. 28594827 PMC5481147

[7] Seemann T. 2014. Prokka: rapid prokaryotic genome annotation. Bioinformatics 30:2068–2069. doi:10.1093/bioinformatics/btu153. 24642063

[8] Laslett D, Canback B. 2004. ARAGORN, a program to detect tRNA genes and tmRNA genes in nucleotide sequences. Nucleic Acids Res 32:11–16. doi:10.1093/nar/gkh152. 14704338 PMC373265

[9] Lowe TM, Eddy SR. 1997. tRNAscan-SE: a program for improved detection of transfer RNA genes in genomic sequence. Nucleic Acids Res 25:955–964. doi:10.1093/nar/25.5.955. 9023104 PMC146525

[10] McNair K, Bailey BA, Edwards RA. 2012. PHACTS, a computational approach to classifying the lifestyle of phages. Bioinformatics 28:614–618. doi:10.1093/bioinformatics/bts014. 22238260 PMC3289917

[11] Garneau JR, Depardieu F, Fortier LC, Bikard D, Monot M. 2017. PhageTerm: a tool for fast and accurate determination of phage termini and packaging mechanism using next-generation sequencing data. Sci Rep 7:8292. doi:10.1038/s41598-017-07910-5. 28811656 PMC5557969

[12] Moraru C, Varsani A, Kropinski AM. 2020. VIRIDIC: a novel tool to calculate the intergenomic similarities of prokaryote-infecting viruses. Viruses 12:1268. doi:10.3390/v12111268. PMC7694805 33172115

[13] Oliveira L, Tavares P, Alonso JC. 2013. Headful DNA packaging: bacteriophage SPP1 as a model system. Virus Res 173:247–259. doi:10.1016/j.virusres.2013.01.021. 23419885

